# Editorial: State-dependent brain computation

**DOI:** 10.3389/fncom.2015.00077

**Published:** 2015-06-23

**Authors:** Petra Ritter, Viktor K. Jirsa, Anthony R. McIntosh, Michael Breakspear

**Affiliations:** ^1^Minerva Research Group Brain Modes, Max Planck Institute for Human Cognitive and Brain SciencesLeipzig, Germany; ^2^Deparment of Neurology, Charité - University MedicineBerlin, Germany; ^3^Bernstein Focus State Dependencies of Learning and Bernstein Center for Computational NeuroscienceBerlin, Germany; ^4^Berlin School of Mind and Brain and Mind and Brain Institute, Humboldt UniversityBerlin, Germany; ^5^Institut de Neurosciences des Systèmes UMR INSERM 1106, Aix-Marseille Université Faculté de MédecineMarseille, France; ^6^Rotman Research Institute of Baycrest Centre, University of TorontoToronto, ON, Canada; ^7^Systems Neuroscience Group, QIMR BerghoferBrisbane, QLD, Australia; ^8^The Royal Brisbane and Woman's HospitalBrisbane, QLD, Australia

**Keywords:** state-dependence, computational neuroscience, brain scales, computational modeling, empirical research

The brain is a self-organizing system, which has evolved such that neuronal responses and related behavior are continuously adapted with respect to the external and internal context. This powerful capability is achieved through the modulation of neuronal interactions depending on the history of previously processed information. In particular, the brain updates its connections as it learns successful versus unsuccessful strategies. The resulting connectivity changes, together with stochastic processes (i.e., noise) influence ongoing neuronal dynamics. The role of such state-dependent fluctuations may be one of the fundamental computational properties of the brain, being pervasively present in human behavior and leaving a distinctive fingerprint in neuroscience data. This development is captured by the present Frontiers Research Topic, “State-Dependent Brain Computation.”

The Research Topic provides an account of prevailing concepts and theories plus recent advances on the role of ongoing brain dynamics—reflecting experiences, global brain states, context and noise—for task-related information processing. Works from the conceptual, experimental and computational-modeling domains are show-cased, focusing on the following two issues: (1) Generative mechanisms of ongoing neuronal dynamics, and (2) Principles of interaction between ongoing dynamics and perceptual or motor processes.

A wide range of spatial and temporal scales encountered in brain dynamics are covered, i.e., from microscopic molecular to macroscopic population dynamics and from fast processes evolving within milliseconds to slow ones taking hours or longer (Table 1). An overview article about state-depended learning exemplifies the need for integration of different scales of processing (Ritter et al., 2015). The role of ongoing alpha oscillations at the microscopic and macroscopic scale for learning is illuminated in Sigala et al. (2014). In this study, the authors present empirical data along with computational models that seek to unveil the underlying principles how oscillations interact with synaptic plasticity. EEG dynamics are also explored in Betzel et al. (2012) where the authors report fast synchronization dynamics—in the range of tens to hundreds of milliseconds—iterating amongst a small set of core networks in the resting brain. The authors suggest that these dynamics may be the neural correlate of resting state BOLD fluctuations. The ability of stochastic dynamic causal modeling (DCM) for fMRI—a neural field formulation of cortical activity—is probed in Daunizeau et al. (2012) where EEG spectral changes are predicted from BOLD signal Fast and high spatial frequency modes as represented in EEG are enslaved by slow and slow spatial frequency modes predominant in fMRI signals. Using an Ising spin model (Deco et al., 2012) demonstrate that the dynamic repertoire of the brain, i.e., different spatio-temporal patterns of functional connectivity, emerges naturally from the neuroanatomical connectivity. It is hypothesized that the scale-free neuroanatomical architecture maximizes the dynamic repertoire and its accessibility in the human brain. Critical slowing caused by dynamical instabilities that are triggered by perception is proposed to enable the brain to process sensory perturbations (Friston et al., 2012). Neuronal oscillator models with surround inhibition were shown to generate bistable spatial patterns of activity (Heitmann et al., 2012) and indicate that state-dependent computations may facilitate rapid switching between motor states, potentially accommodating high speed rather than precision responses. The cross-frequency coupling present in empirical EEG was systematically simulated in a full human brain network model of coupled neuronal oscillators (Jirsa and Muller, 2013) for eyes-open and eyes-closed states of rest condition and the theoretical implications for state-dependent processing discussed. Distinct brain states linked to motor and perceptual visuo-spatial working memory and accompanying specific mental processes are characterized as spatio-temporal functional connectivity patterns in EEG (Protopapa et al., 2014).

**Table 1:** **Different facets of state-dependent brain computation are illuminated in the present Research Topic**.

**Scale**	**Article**	**Animal**	**Function**	**States**	**Empirical**	**Model**
Micro	Banerjee et al., 2012	Macaque	Arm movement/reaching task	LFP	Spike trains, LFPs	Parametric model
	Fernandez-Ruiz and Herreras, 2013	Rat	Rest, stimulation	LFP	LFP	–
	Humble et al., 2012	–	Memory	Spike trains	–	Spiking neuron models and STDP
	Mark and Tsodyks, 2012	Rat	Spontaneous, sensory stimulations	Different synchrony levels	–	Wilson-Cowan rate model, IF-neurons, Realistic neuronal network with clustered architecture
	Palma et al., 2012	–	Memory	Neuromodulation	–	Spiking recurrent networks
	Quilichini and Bernard, 2012	Rat	T-maze	Neuromodulators	Firing pattern	–
Bridging micro to macro	Ritter et al., 2015	Human, macaque, rat, *in vitro*	Learning, rest	Oscillatory LFP EEG BOLD	Yes	Yes
	Sigala et al., 2014	Human/young adults	Learning, rest	Oscillatory LFP EEG BOLD	Yes	Yes
Macro	Betzel et al., 2012	Human	Rest	EEG, 10–100 ms	EEG	–
	Daunizeau et al., 2012	Human/patients with epilepsy	Rest, interictal activity	EEG BOLD	EEG, BOLD	Neuronal field model/stochastic DCM, Heuristic model
	Deco et al., 2012	Human	Rest		BOLD	Ising spin model
	Heitmann et al., 2012	Human	Motor	LFP/EEG	–	Neuronal oscillator model
	Jirsa and Muller, 2013	Human	Rest, eyes-closed, eyes-open	EEG	EEG	generic oscillator equations derived from coupled full brain network
	Miller et al., 2012	Human	Rest and task/stimulation	Band-limited EEG rhythms amplitude modulation	EEG	–
	Protopapa et al., 2014	Human	Motor and visuo-spatial working memory	EEG functional connectivity	EEG	–
Not specified	Friston et al., 2012	Human, bird	Sensory stimulation, bird song	Behavioral, simulated neuronal dynamics	–	Generalized Bayesian filtering, generic generative model

On the microscopic scale, spike trains and local field potentials (LFP) dynamics are set in relation (Banerjee et al., 2012) using parametric models with the goal to decode those signals and infer related behaviors. In Fernandez-Ruiz and Herreras (2013) it is pointed out that LFP's are highly variable over time and have flexible spectrums, i.e., the notion of periodic oscillations commonly used to describe brain activity is questioned. These authors propose a method to de-mix LFPs of different sources to determine the true degree of periodicity—a prerequisite for a mechanistic understanding of information transfer in the brain. In a spiking neuronal model with STDP (Humble et al., 2012) demonstrate that simple networks of laterally connected excitatory neurons can self-organize into spatio-temporal pattern recognizers. The potential for representations of more complex nested patterns which implies stronger computational memory capabilities is raised. The flow of information depends on the degree of network synchrony (Mark and Tsodyks, 2012) and an intermediate degree of synchrony is most beneficial for information transfer. The question whether rhythmic entrainment represents a general mechanism of computation in the brain is raised and ways are pointed out how to address this question through empirical work in the future (Miller et al., 2012). The theoretical impact of neuromodulation on memory formation in spiking recurrent cortical networks is systematically evaluated (Palma et al., 2012). In a perspective article, a systematic account is provided how intrinsic properties of neurons and neuromodulation relates to firing patterns, functional correlations and behavior in rats (Quilichini and Bernard, 2012).

The degree of abstraction in the modeling work presented in this Research Topic varies tremendously, ranging from simplified but biophysically plausible network models to highly detailed neuron models. By placing the different mathematical and empirical aspects in this mutual context, this Research Topic aims to elucidate the principle mechanisms of state-dependent neuronal processing. Developing a framework to link the multiple principles together is arguably the most pressing challenge. With The Virtual Brain (thevirtualbrain.org) simulation framework (Ritter et al., 2013; Sanz Leon et al., 2013) we hope to contribute to this endeavor by enabling researchers to use multiple modeling approaches in a unified framework ensuring reproducibility and comparability of results.

## Conflict of interest statement

The authors declare that the research was conducted in the absence of any commercial or financial relationships that could be construed as a potential conflict of interest.
